# Signatures of selection in pleiotropic genes involved in insect neuronal and immune systems

**DOI:** 10.1093/g3journal/jkag223

**Published:** 2026-08-13

**Authors:** Sowmya Senthilkumar, Reese A Martin, Ann T Tate

**Affiliations:** Department of Biological Sciences, Vanderbilt University, Nashville, TN 37232, United States; Department of Biological Sciences, Vanderbilt University, Nashville, TN 37232, United States; Department of Biological Sciences, Vanderbilt University, Nashville, TN 37232, United States; Evolutionary Studies Initiative, Vanderbilt University, Nashville, TN 37240, United States

**Keywords:** neuroimmunology, neurodegenerative, evolvability, adaptation, evolutionary statistics

## Abstract

Pleiotropy, where a single gene contributes to multiple biological functions, plays a central role in shaping evolutionary constraints. The nervous and immune systems are tightly integrated both functionally and genetically, but it is not clear whether pleiotropy constrains adaptation in each system or contributes to disease. Building on evidence that genes that are pleiotropic between development and immunity in *Drosophila melanogaster* evolve slower than immune-specific genes, we tested whether pleiotropic neuro-immune genes likewise evolve under stronger purifying selection compared to genes functioning solely in neuronal or immune processes, and whether such evolutionary constraint is associated with human neurological disease. We identified immune, neuronal, and neuro-immune genes in *D. melanogaster* using gene ontology (GO) annotations, estimated patterns of evolutionary constraint across 12 *Drosophila* species using dN/dS ratios, and investigated the breadth of their expression across developmental stages as a complementary proxy for pleiotropy. We further curated associations between the human orthologs for these genes and neurological disease and tested whether their evolutionary statistics and pleiotropic status predicted disease involvement. We found that pleiotropic genes exhibited significantly lower dN/dS values and were less stage-specific than nonpleiotropic immune genes. Slower-evolving genes were more likely to be associated with human neurological diseases but this pattern was strongest for nonpleiotropic neuronal genes, suggesting that pleiotropy alone is not the strongest predictor of disease. Our results therefore indicate that dN/dS could be a stronger predictor of disease association than pleiotropy, raising new questions about the maintenance of pleiotropy in evolutionarily dynamic physiological systems.

## Introduction

Pleiotropy, the phenomenon in which a single gene influences multiple traits, has long been recognized as a central feature of evolution because it constrains how organisms respond to natural selection (Orr 2000; Wagner et al. 2008; Paaby and Rockman 2013). Pleiotropy can range from a gene affecting multiple phenotypic traits to one participating in several biological processes or physiological systems (Paaby and Rockman 2013; Fraïsse et al. 2019). Because mutations in pleiotropic genes can influence multiple functions simultaneously, adaptive changes that benefit one process may compromise another, often resulting in stronger purifying selection and reduced evolutionary flexibility (Orr 2000; Wagner et al. 2008; Fraïsse et al. 2019).

As with most physiological systems that have distinct developmental trajectories and functions, nervous and immune systems have traditionally been studied independently from one another. However, growing evidence hints at substantial molecular and cellular interplay between these systems. For example, *Xenopus* embryos lacking normal brain development exhibited significantly reduced survival following bacterial infection compared with control embryos (Herrera-Rincon et al. 2020), revealing that very early brain development plays a central role in innate immunity (Kraus et al. 2021). Neurons release immunomodulatory neurotransmitters that can either amplify or suppress immune responses, while immune cells produce neuroactive cytokines that influence neuronal function (Kipnis 2018). West Nile virus, for example, disrupts the balance between the immune and nervous systems by activating genes with multiple roles in each system, suggesting that viral infections may impair neurotransmission by triggering immune responses that negatively affect neuronal function (Maximova et al. 2021). Additionally, both the nervous and immune systems undergo extensive developmental regulation, and genes expressed across multiple life stages often experience strong evolutionary constraint (Liao and Zhang 2006).

This functional overlap poses interesting questions about the evolutionary dynamics of pleiotropic immune and neuronal genes. After all, the immune system is under pressure to rapidly adapt to pathogens, and many immune genes show elevated levels of positive or balancing selection (Sackton et al. 2007; Obbard et al. 2009; Williams et al. 2023). While some neuronal pathways show lineage-specific diversification consistent with adaptation (Tuller et al. 2008; Yin and Yuan 2015), many genes expressed in neural tissues evolve under strong purifying selection (McCoy and Fire 2024). For example, arylsulfatase A and galactocerebrosidase are lysosomal enzymes required for the metabolism of a major lipid component of the myelin sheath that insulates axons and enables rapid nerve impulse transmission. Their orthologs are highly conserved across vertebrates and exhibit strong purifying selection, consistent with the genes essential for white-matter function (Luetzen et al. 2025). Recent work suggests that immune genes with developmental roles evolve more slowly than those dedicated solely to immunity (Williams et al. 2023), raising the possibility that neuro-immune pleiotropy similarly constrains immune gene evolution. This idea is supported by the fact that many neuronal genes have critical developmental functions, suggesting they may be constrained similarly to developmental genes. If true, this would suggest that the adaptive potential of immune defenses is not solely dictated by pathogen pressure but also by the evolutionary constraints imposed by their additional roles in neural processes.

The relevance of this pleiotropic conflict extends beyond basic biology into the clinic. Many genes associated with neurological diseases such as Alzheimer's and Parkinson's also participate in immune defense (Bellou et al. 2020; Provenzano and Deleidi 2021). For example, a patient-derived BCL11B mutation (N441 K) causes combined immune and neurological defects. In mice, the equivalent mutation (N440 K) led to both abnormal NK/ILC1-like immune cell development in the thymus and reduced cortical neurons (Okuyama et al. 2024). Hyperactive immune responses, moreover, protect against infection but can trigger neuroinflammation, accelerating neurodegeneration (Provenzano and Deleidi 2021). Understanding how the shared genes and pathways navigate competing pressures could reveal why certain neurological disorders persist in populations despite their costs to health and fitness.

Many of the mechanisms controlling neural development and immune activation are conserved between insects and mammals (Lye and Chtarbanova 2018). The extensive genetic and genomic data and tools available for the fruit fly *Drosophila melanogaster* make it an excellent model organism to explore how pleiotropy influences selection pressures and organismal phenotypes associated with infection and neurodegenerative diseases in humans (Lye and Chtarbanova 2018). We analyzed values of dN/dS, the ratio of nonsynonymous to synonymous substitutions, for pleiotropic immune-neuronal genes across 12 *D. melanogaster* species to test whether nervous system functions impose evolutionary constraints on immune adaptation. We predicted that immune genes with neuronal roles would have lower dN/dS ratios than immune-specific genes, reflecting stronger purifying selection. We further predicted that pleiotropic genes would have more associations with neurological disorders compared to nonpleiotropic genes after controlling for evolutionary rate, as their constrained evolution may reflect strict roles in neuronal development and maintenance. Support for these predictions would shed light on how pleiotropy shapes the molecular evolution of interconnected biological systems more broadly as well as trade-offs between immunity and nervous system maintenance.

## Materials and methods

### Categorization of nonpleiotropic and pleiotropic immune and neuronal genes

We extracted immune-related and neuronal gene lists for *D. melanogaster* from FlyBase by manually collecting GO annotated genes tagged with the “immune system process” (GO: 0002376) or “neuron development” (GO: 0048666) terms, respectively. We selected broad, high-level GO terms rather than highly specific descendant terms because they capture a wide range of genes involved in each physiological system. Although alternative GO terms could have been selected, our goal was to identify genes broadly associated with immune and neuronal functions rather than genes involved in specific immune or neuronal sub-processes. Because our neuronal classification included genes annotated with neuron development GO terms, the pleiotropic category encompasses genes involved in both neurodevelopment and mature neuronal function. Rather than separating developmental and functional neuronal roles, our goal was to examine the evolutionary consequences of genes broadly shared between the nervous and immune systems. From these, we identified 724 neuronal genes, 584 immune genes, and 96 genes with overlapping functions, which we defined as pleiotropic. In this study, we adopt a system-level definition of pleiotropy and focus specifically on genes annotated with functions in both nervous system and immune system processes. Binning genes into a “non-pleiotropic” class does not mean they have *no* pleiotropic function; after all, most genes are likely pleiotropic if enough traits are considered (Boyle et al. 2017); these genes simply do not exhibit pleiotropy between our 2 major systems of interest (see additional discussion of this approach in Williams et al. 2023). The full list of genes in each group is included in Supplementary Table 1 in Supplementary File 1.

### Compiling sequences for analysis

We followed the protocol established in Williams et al. (2023), where a subset of the *Drosophila* clade was used to determine the rate of evolution of genes that were pleiotropic between developmental and immune processes. First, we obtained coding sequences (CDSs) for *Drosophila melanogaster* using the FlyBase Sequence Downloader tool with FlyBase gene IDs. From an archived FlyBase version (FB2017_05), we retrieved ortholog lists for 12 Drosophila species (*D. ananassae, D. erecta, D. grimshawi, D. mojavensis, D. persimilis, D. pseudoobscura, D. sechellia, D. simulans, D. virilis, D. willistoni*, and *D. yakuba*). The previous study (Williams et al. 2023) demonstrated that the main results were relatively insensitive to the use of this broader species set vs smaller subgroups despite the risk of saturated dS, so we chose to repeat the 12 species approach here for consistency. We downloaded CDSs for each gene of interest across species using the archived FlyBase Sequence Downloader tool. Using scripts adapted from the Supplementary materials of that study, we parsed FlyBase sequence IDs for each species.

We aligned sequences in codon space and trimmed them using Gblocks v0.91b (Castresana 2000). When we encountered paralogs, we excluded those genes from alignment because duplicated genes can experience divergent evolutionary trajectories following duplication, potentially violating assumptions of orthology-based dN/dS comparisons and inflating estimates of evolutionary divergence. We retained only genes with ≥2 high-quality orthologous CDSs for downstream analysis, resulting in 545 neuronal, 428 immune, and 77 pleiotropic curated alignments. We used these filtered gene sets (rather than the initial GO-derived lists) for all evolutionary and biological-process count analyses described below.

### Biological counts as a measure of pleiotropy

To characterize the prevalence of pleiotropy within the filtered gene sets, we quantified the number of unique GO biological process annotations per gene. For each gene included in the evolutionary analyses, GO analysis for each gene were retrieved using the FlyBase “ID Validator” tool, and biological process annotations were extracted from the resulting hit lists. The number of biological processes for each gene were then summarized for each functional category (neuronal, immune, pleiotropic; Supplementary Table 2 in Supplementary File 1). The biological process counts for each gene ID are listed in Supplementary Table 3 in Supplementary File 1.

### Calculating dN/dS values using PAML

The dN/dS ratio, which compares nonsynonymous to synonymous substitutions, served as our measure of evolutionary constraint. A dN/dS > 1 indicates positive selection driving amino acid changes, while a lower ratio reflects purifying selection preserving protein structure (Jackowiak et al. 2014). Relatively higher dN/dS ratios still suggest that a gene is experiencing stronger positive selection, or at least relaxed purifying selection, compared to others. We used these sequence files to calculate the dN/dS ratios by running them through codeml site model M0 in PAML (v 4.10.7). Our PAML analysis successfully processed all gene lists (539 neuronal genes, 420 immune genes, and 74 pleiotropic genes). In addition to per-gene analyses, we generated concatenated alignments for each gene category to estimate overall category-level dN/dS values using PAML model M0. Because these variables were non-normally distributed, we performed Kruskal–Wallis tests followed by post hoc Dunn tests in R (v2026.01.0) to compare dN/dS values. The dN/dS values for each gene in each category are listed in Supplementary Table 4 in Supplementary File 1.

### RNA-seq expression data acquisition and preprocessing

RNA-seq gene expression data were obtained from the modENCODE developmental expression datasets curated in FlyBase. Specifically, we downloaded reads per kilobase per million ([RPKM] mapped reads) values from the FlyBase gene_rpkm_report_fb_*.tsv.gz files, which compile normalized RNA-seq expression estimates across developmental stages for *Drosophila melanogaster* genes. For each gene class (pleiotropic, nonpleiotropic neuronal, and nonpleiotropic immune), we filtered the full FlyBase expression matrix to retain only genes belonging to the corresponding category (Supplementary Tables 5 to 8 in Supplementary File 1). Gene identifiers were matched using FlyBase gene IDs (FBgn). Expression values were extracted for 3 representative developmental stages: larval stage 1 (L1), pupal stage 5 (P5), and adult male 1 d post-eclosion (AdM_Ecl_1days).

Embryonic stages were excluded from the main analysis because they are represented by multiple short time windows (eg 0 to 2 h, 2 to 4 h, etc.), which would disproportionately weight embryonic expression and artificially depress τ values when aggregated into a single developmental category. Restricting the analysis to larval, pupal, and adult stages allowed for balanced comparison of expression specificity across major post-embryonic developmental transitions, but results that include the embryonic stage are presented in the Supplementary Materials.

### Calculation of gene expression specificity (τ)

To quantify developmental expression specificity, we calculated the τ (tau) metric as originally described by Yanai et al. (2005). Tau provides a normalized measure of the specificity of gene expression across tissues or developmental stages, ranging from 0 (broad expression across all stages) to 1 (highly stage-specific expression).

For each gene, τ was calculated across the 3 developmental stages using the following equation:


$$\tau =\;\overset{n}{\underset{\;j=1}{\sum }}\;\begin{array}{c} \frac{1-\frac{\log\;(A_{j})}{\log\;(A_{max})}}{n-1} \end{array}$$

where *n* represents the number of stages, *A*ⱼ the expression level (RPKM) of the gene at stage/tissue j, and *A*_max_ is the maximum expression value of that genes across all stages.

Prior to log transformation, a pseudocount of 1 was added to all RPKM values to avoid undefined logarithms for genes with zero expression at one or more stages. Zero-expression observations were retained in the dataset and were not removed; the pseudocount was introduced only for the calculation of τ values and did not alter gene inclusion in downstream statistical analyses. Tau values were computed independently for each gene, resulting in a gene-level distribution of τ values for each gene class. Differences in τ distributions among pleiotropic, nonpleiotropic neuronal, and nonpleiotropic immune gene classes were assessed using a Kruskal–Wallis rank-sum test.

When the Kruskal–Wallis test indicated a significant overall effect, post hoc pairwise comparisons between gene classes were performed using Dunn's test. Resulting *P*-values were adjusted for multiple comparisons using the Benjamini–Hochberg false discovery rate (FDR) correction. The specific tau values for each gene are listed in Supplementary Table 9 in Supplementary File 1.

### Gene ontology cellular component enrichment analysis

GO enrichment analyses were performed using PANTHER, focusing specifically on the Cellular Component (GO-CC) ontology. Enrichment results were reported as fold enrichment values, representing the ratio of observed gene counts in a given GO category relative to the expected number based on background frequency. The *P* values were corrected for multiple testing using FDR correction as implemented by PANTHER. Fold enrichment values were log_2_ transformed and log_2_ fold enrichment = 0 corresponds to a fold enrichment of 1. To facilitate comparison across gene categories while minimizing visual clutter, a shared set of GO Cellular Component terms was selected for plotting. Specifically, for each gene category, the top N GO terms (*N* = 10) were ranked by FDR-adjusted *P* value, prioritizing the most statistically significant enrichments. The union of these top-ranked terms across the 3 gene categories was used to define the final set of GO terms displayed, resulting in approximately 22 GO terms after accounting for overlap among categories. Because the gene sets were initially defined using broad GO biological process annotations, we expected enrichment analyses to recover more specialized descendant terms within these functional categories. The GO enrichment analysis is interpreted not as an independent validation of gene classification, but rather as a means of characterizing and comparing the cellular localization of the resulting gene sets. Complete enrichment statistics, including GO identifiers, gene counts, and FDR values, are provided in the Supplementary Tables 10 to 12 in Supplementary File 1.

### The relationship between dN/dS and neuronal diseases

To examine whether dN/dS was associated with neuronal disease, we annotated each gene in our dataset for known human neurological disease associations. We used the FlyBase ID Validator tool to retrieve human ortholog information and associated Human Disease Ontology annotations for each *D. melanogaster* gene. For every gene, we manually screened all annotations for relevance to neurological or neurodevelopmental disease categories, using keywords including but not limited to: *neurodevelopmental disorder*, *neurodegenerative disease*, *motor neuron disease*, *Parkinson disease*, *Alzheimer disease*, *ataxia*, and *intellectual disability*. Based on these annotations, each gene was assigned a binary neuronal-disease variable, where 1 indicated at least one associated neurological disease term in its human ortholog(s), and 0 indicated the absence of any such disease annotation.

To examine the relationship between dN/dS ratio and neuronal disease association, we fit a binomial logistic regression model with neuronal disease status as the response variable and both gene class (pleiotropic vs nonpleiotropic) and gene-specific dN/dS as predictors. Models were fit in R using the glm function with a binomial distribution. Test statistics and associated *P*-values were obtained from the resulting analysis.

The full step-by-by step protocol, including scripts, can be explored here:

https://dx.doi.org/10.17504/protocols.io.rm7vz49d8lx1/v1

## Results

### Pleiotropic genes are involved in more biological processes than nonpleiotropic immune and neuronal genes

To determine if the genes we identified as pleiotropic between immunity and the nervous system were in general more pleiotropic than purely immune or nervous genes, we counted the total number of unique biological processes associated with each gene in our study (Supplementary Fig. 1). We found that pleiotropic genes are associated with significantly more biological processes than nonpleiotropic immune (*P*.adj = <0.0001) and neuronal (*P*.adj < 0.0001) genes (Fig. 1). There was no significant difference in biological process counts between the nonpleiotropic groups (*P*.adj = 0.06).

**Fig. 1.**
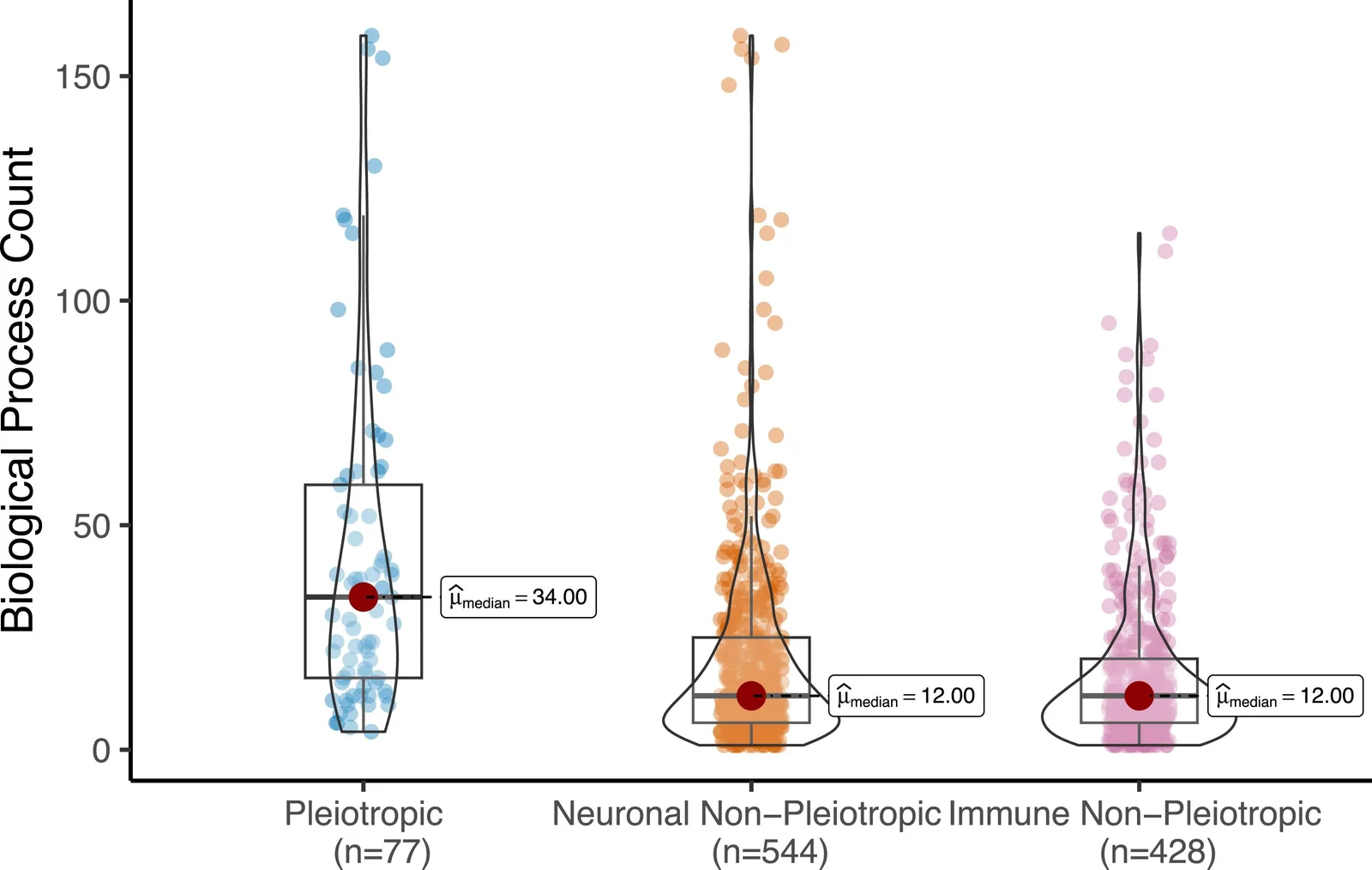
Number of biological processes associated with each class of genes. Each gene was categorized into a class based on its annotated function(s). The number of unique annotated GO biological processes was determined for each gene. These counts reflect the number of functional roles a gene plays in various biological processes and are shown on the *y*-axis. The violin plots show the distribution density of biological process counts across genes in each category. Individual points represent individual genes. Post hoc pairwise Dunn tests were used to calculate pairwise comparisons, and *P* values were adjusted for false discovery rate (Benjamini–Hochberg FDR).

### Pleiotropic and nonpleiotropic gene classes show distinct patterns of cellular component enrichment

We performed GO cellular component enrichment analyses on pleiotropic, nonpleiotropic neuronal, and nonpleiotropic immune gene sets and compared patterns of enrichment across gene classes (Fig. 2). Across the top significantly enriched GO cellular component terms for each gene set, pleiotropic genes showed enrichment for a wide range of broadly defined cellular structures, including *cell body*, *cell projection*, *cytoplasmic vesicle*, *intracellular vesicle*, and *plasma membrane-bounded cell projection* (Fig. 2). In contrast, nonpleiotropic neuronal genes exhibited stronger enrichment for neuron-specific compartments, such as *axon*, *somatodendritic compartment*, *neuron projection*, and *neuronal cell body*, consistent with functional specialization within the nervous system. Nonpleiotropic immune genes were enriched for a distinct set of cellular components, including *extracellular region*, *extracellular space*, *perivitelline space*, and immune-related complexes such as the *calcineurin complex*, indicating specialization toward immune-associated cellular contexts.

**Fig. 2.**
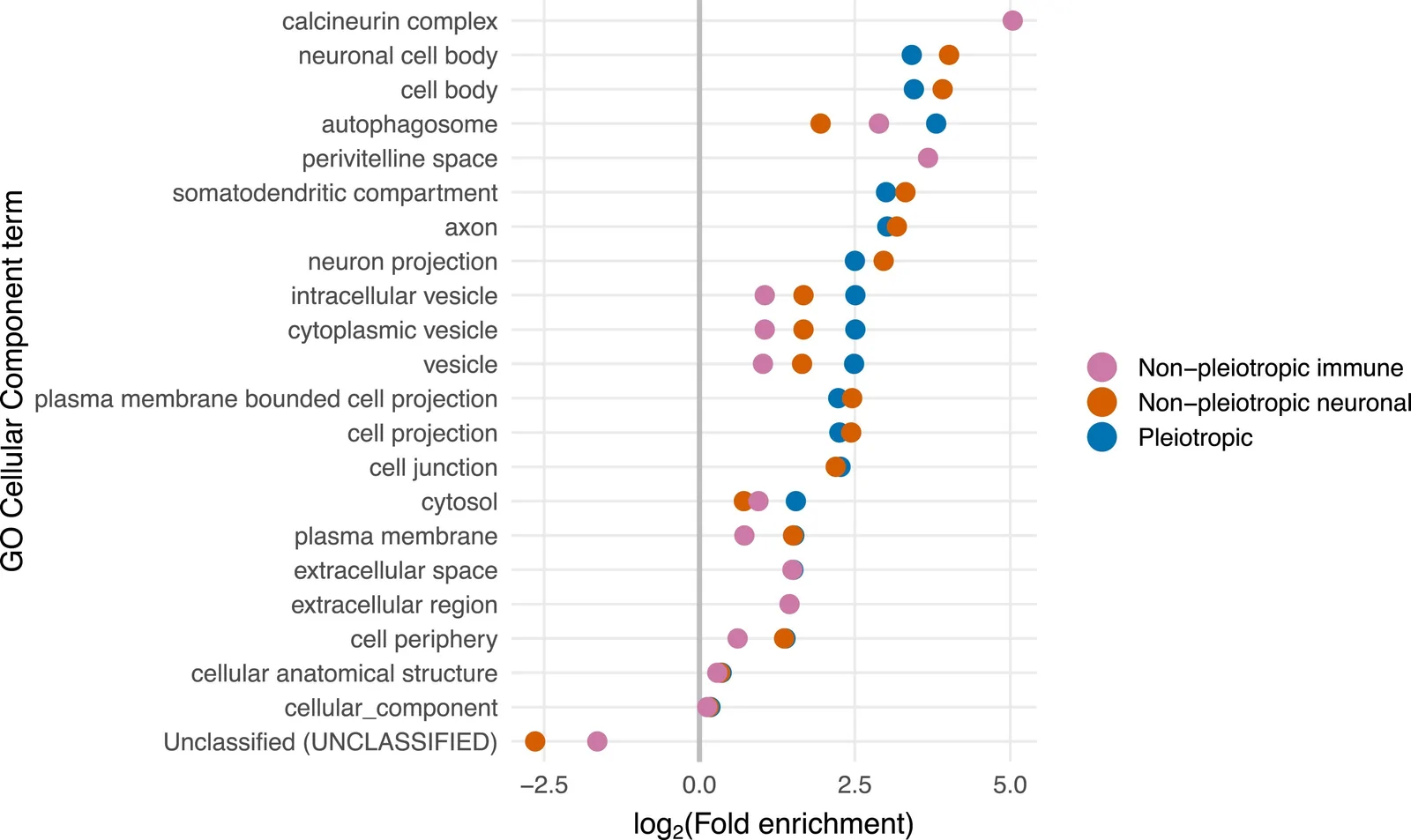
GO cellular component enrichment analysis comparing pleiotropic, nonpleiotropic neuronal, and nonpleiotropic immune gene sets. The dot plot displays the log_2_ fold enrichment for significantly enriched GO cellular component terms. Each row corresponds to a GO Cellular Component term, and each dot represents a statistically significant enrichment for a given gene category (FDR ≤ 0.05). Dot color denotes gene category. Only GO terms that were among the top FDR-significant terms across gene categories are shown. Absence of a dot indicates that the GO term was not significantly enriched in that gene category.

Several GO terms were shared across gene classes but differed in the magnitude of enrichment, with pleiotropic genes generally showing moderate enrichment across many cellular components, whereas nonpleiotropic gene classes displayed stronger enrichment in fewer, more specialized compartments.

### Pleiotropic genes evolve more slowly than nonpleiotropic immune and neuronal genes

To compare evolutionary rates of pleiotropic genes compared to the nonpleiotropic genes, we ran PAML model M0 with concatenated lists of pleiotropic and nonpleiotropic genes. The PAML model on the gene lists gave median dN/dS ratios of 0.0516, 0.0592, and 0.0748 for pleiotropic genes, neuronal genes, and immune genes, respectively.

Pleiotropic genes evolve significantly more slowly than nonpleiotropic immune genes (*P*.adj = 7.87e−04; Fig. 3). There was no significant difference between the pleiotropic genes and nonpleiotropic neuronal genes (*P*.adj = 0.29), but nonpleiotropic immune genes did have significantly higher dN/dS values than nonpleiotropic neuronal genes (*P*.adj = 8.19e−06).

**Fig. 3.**
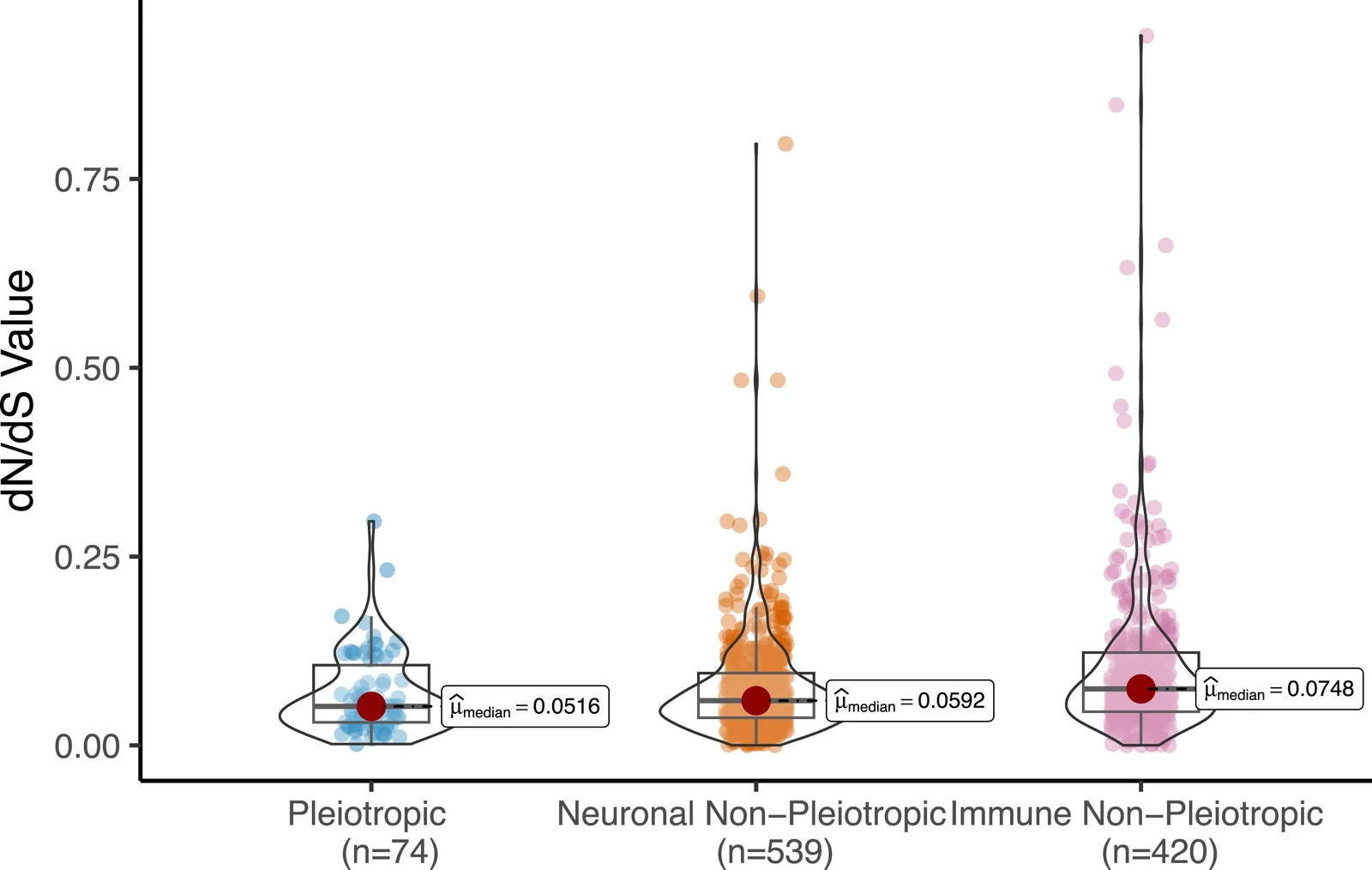
Violin plot showing the distribution of dN/dS ratios for 3 gene categories: pleiotropic, nonpleiotropic neuronal, and nonpleiotropic immune. Each violin plot represents the distribution of dN/dS values for genes in a given category. The width of the violin corresponds to the density of genes at different dN/dS values. Higher dN/dS values suggesting positive or relaxed purifying selection and lower dN/dS values suggest stronger purifying selection. Post hoc pairwise Dunn tests were used to calculate pairwise comparisons, and *P* values were adjusted for false discovery rate (Benjamini-Hochberg FDR).

### Pleiotropic genes are more broadly expressed across life stages than nonpleiotropic ones

Pleiotropic genes are hypothesized to be more broadly expressed across stages than nonpleiotropic immune genes, reflecting their role in multiple or housekeeping functions throughout life (Yanai et al. 2005; Williams et al. 2023). To test this hypothesis, we assessed the uniformity of gene expression across larval, pupal, and adult stages in *D. melanogaster* by comparing the distribution of tau values (τ) for each gene in each gene class (Supplementary Fig. 2). Lower τ values indicate broader expression across life stages. Genes in the nonpleiotropic immune class were significantly more stage-specific than nonpleiotropic neuronal genes (*P*.adj = 2.96e−03) and pleiotropic genes (*P*.adj = 0.04; Fig. 4). Pleiotropic genes and nonpleiotropic neuronal genes had similar stage specificity values (*P*.adj = 0.59; Fig. 4). All significant effects disappeared when the embryonic stage was added to the analysis, due to an extreme excess in the expression of embryo-only genes (Supplementary Fig. 3).

**Fig. 4.**
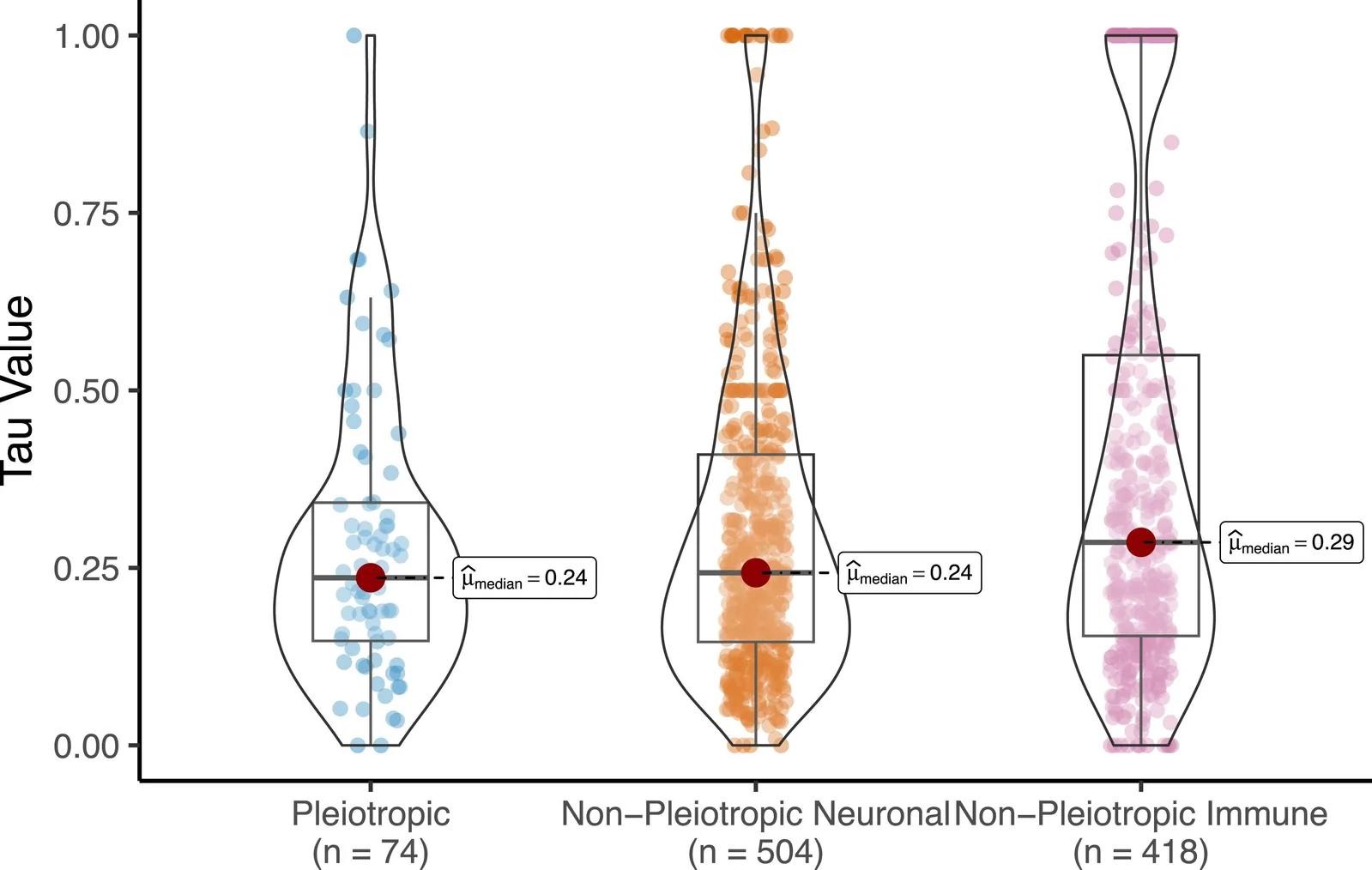
Gene expression specificity (tau) compared across larval, pupal, and adult stages for pleiotropic and nonpleiotropic gene classes. Tau values represent the gene expression specificity and range from 0 (broadly expressed across stages) to 1 (specific to a stage). Each violin represents the density distribution of tau values for a gene category. Individual dots represent tau values for each gene within the category. Median tau values are labeled for each group. The *P* values were statistically obtained by the Kruskal–Wallis test. Post hoc pairwise Dunn tests were used to calculate pairwise comparisons, and *P* values were adjusted for false discovery rate (Benjamini–Hochberg FDR).

### Genes under stronger purifying selection are more strongly associated with human neuronal diseases

To examine the relationship between evolutionary constraint and neuronal disease association, we performed a binomial logistic regression with neuronal disease status as the response variable and both dN/dS and gene class (pleiotropic vs nonpleiotropic) as predictors (Fig. 5). Across all genes, dN/dS was a significant predictor of disease association. Specifically, dN/dS showed a strong negative relationship with the probability of neuronal disease involvement (Supplementary Table 13 in Supplementary File 1; coefficient estimate = −13.36, SE = 3.61, *P* = 0.0002), indicating that genes evolving under stronger purifying selection were more likely to be associated with neurological disease.

**Fig. 5.**
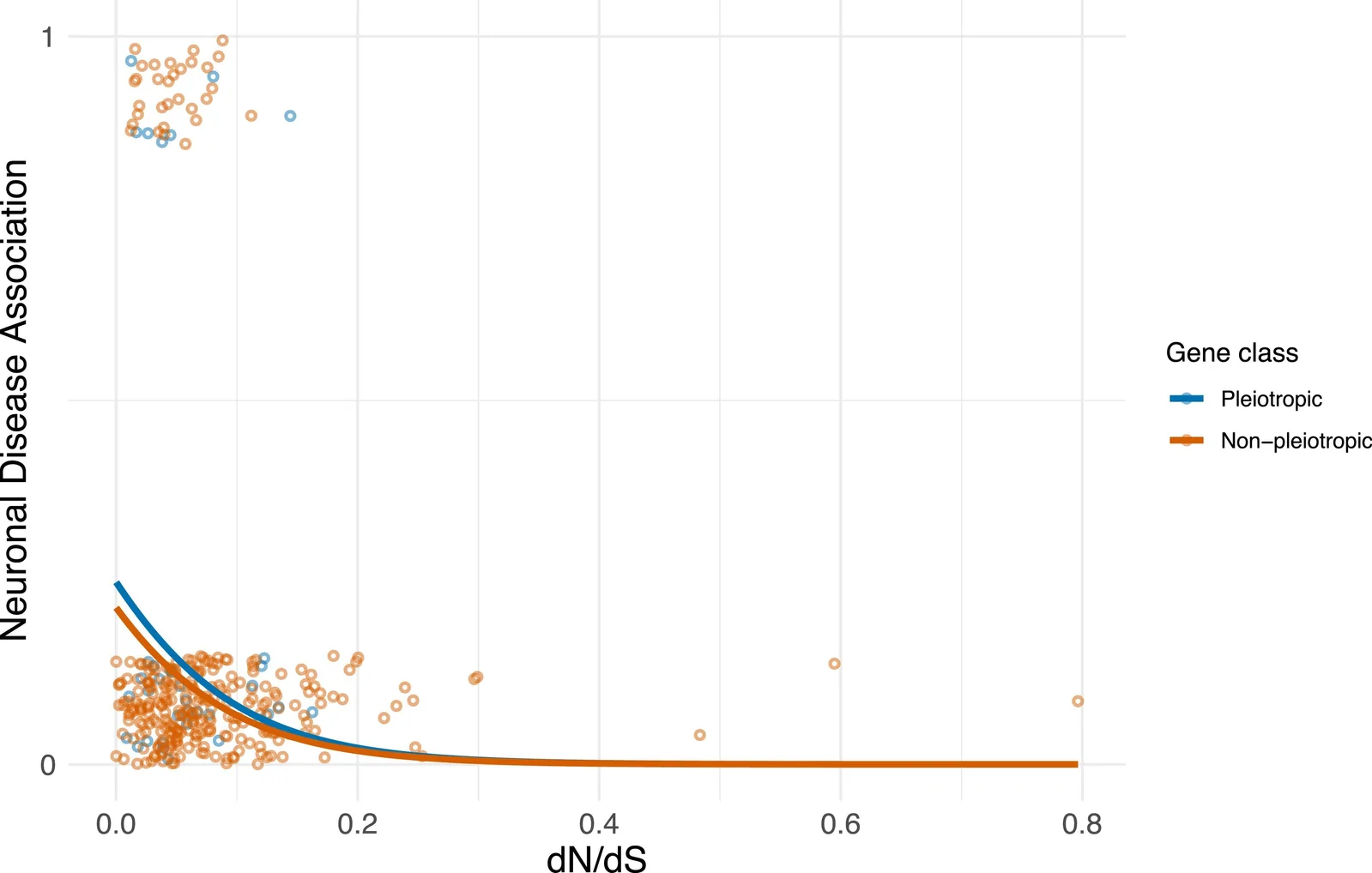
Binomial distribution showing the relationship between dN/dS ratio values and neuronal disease association for individual genes in pleiotropic and nonpleiotropic gene classes. Logistic regression curves are shown for pleiotropic genes (blue) and nonpleiotropic genes (orange). The *x*-axis represents dN/dS values, and the *y*-axis represents the binary outcome of neuronal disease association (0 = no association, 1 = associated), as determined from human disease annotations linked to each gene's human ortholog in FlyBase. Each point represents an individual gene, and the fitted curves show the predicted probability of neuronal disease association across increasing dN/dS values for each gene class. All dots are either 0 or 1 on the *y*-axis but are jittered for ease of viewing.

In contrast, gene class was not a significant predictor after accounting for dN/dS ratios (coefficient estimate = −0.20, SE = 0.37, *z* = −0.53, *P* = 0.60). The fitted logistic curves for pleiotropic and nonpleiotropic genes show similar trends, with both gene classes exhibiting decreasing probabilities of disease association as dN/dS increases.

## Discussion

In this study, we tested the hypothesis that pleiotropy constrains the evolution of genes involved in both immune and neuronal systems relative to genes that function in only one of the 2 processes. Our results suggest that pleiotropic genes do evolve more slowly than nonpleiotropic immune genes but evolve at a similar rate compared to nonpleiotropic neuronal genes. Our results further suggest that neurological disease associations are more strongly linked to genes evolving under stronger purifying selection, and that this relationship is better explained by evolutionary constraint than by pleiotropy alone.

The pleiotropic genes in our study were associated with significantly more GO biological processes than either of the nonpleiotropic groups (Fig. 1). This estimate, which includes all GO biological processes and not just neuronal and immune ones, provides complementary support that these genes are more pleiotropic overall than those in the nonpleiotropic categories. The highest median dN/dS value observed in this study was associated with the nonpleiotropic immune genes. This aligns with prior evidence that many immune genes, particularly those without pleiotropic constraints, undergo rapid adaptive evolution (Williams et al. 2023). The median dN/dS values for the nonpleiotropic neuronal and nonpleiotropic immune genes were statistically different from each other, suggesting that at least some neuronal nonpleiotropic genes evolve under less constraint than their pleiotropic counterparts. Previous work suggests that genes highly expressed in cortical regions of the brain have stronger purifying selection than genes enriched in subcortical regions (Tuller et al. 2008), providing a biological explanation for our observation of a large spread in the distribution of dN/dS values in nonpleiotropic neuronal genes (Fig. 3). Consistent with this interpretation, nonpleiotropic neuronal genes also show strong enrichment in highly specialized neuronal compartments such as axons and somatic and dendritic regions (Fig. 2), suggesting that cellular compartmentalization may further influence variation in evolutionary constraint.

The statistical relationship between neuronal pleiotropy and evolutionary constraint observed in this study builds directly on the developmental pleiotropy framework established by Williams et al. (2023), which serves as the foundation for our analysis. That study demonstrated that developmental genes, as well as genes with dual roles in immunity and development, evolve more slowly and experience stronger purifying selection than nonpleiotropic immune genes. Consistent with this framework, just as developmental genes are predicted to be under purifying selection to ensure a faithful developmental program, the stronger evolutionary constraint of pleiotropic neuronal genes in our study may arise from their conserved effects on neural network maintenance (McCoy and Fire 2024). These findings are also consistent with theoretical expectations from the cost-of-complexity framework, which predicts that mutations affecting multiple biological functions are less likely to be beneficial because they increase the probability of deleterious effects on one or more traits (Orr 2000; Wagner et al. 2008). Accordingly, neuro-immune pleiotropic genes may experience stronger evolutionary constraint because adaptive changes must preserve function across both physiological systems. While previous work has examined broad patterns of pleiotropy across biological traits (Fraïsse et al. 2019; Martin and Tate 2025), our study extends this framework by examining the evolutionary consequences of pleiotropy within the specific context of neuro-immune interactions.

Notably, neuro-immune pleiotropic genes show evolutionary patterns that more closely resemble other neuronal genes than immune genes. The lower dN/dS values observed in pleiotropic neuronal genes are consistent with stronger purifying selection compared to nonpleiotropic immune genes (Fig. 3). This may relate to functional constraints on immunity that protects delicate neural tissue from potentially damaging inflammation (Buckley and McGavern 2022). Although once thought to lack immune function, neurons and glial cells do express many immune-related proteins in restricted spatial and temporal patterns consistent with nonimmunological roles. For instance, major histocompatibility complex class I (MHCI) proteins, which are central to adaptive immunity, are also expressed in neurons, where they contribute to neural signaling and plasticity (Elmer and McAllister 2012). Consistent with this broader pattern, pleiotropic genes in our dataset were enriched for neuronal cellular compartments such as axons and neuronal cell bodies (Fig. 2), supporting the idea that immune-related genes with essential neuronal functions experience stronger evolutionary constraint. Therefore, although different GO enrichment parameters may modestly alter gene assignment to a category, we expect the overall pattern of stronger evolutionary constraint among neuro-immune pleiotropic genes to remain consistent.

The observation that pleiotropic genes are more broadly expressed across developmental stages than nonpleiotropic immune genes is consistent with previous results on immune-developmental pleiotropy (Williams et al. 2023) and provides insight into the roles these genes play throughout the organism's development (Fig. 2). Previous work has highlighted that genes involved in multiple biological processes often display conserved, widespread expression due to their essential roles in core cellular functions (Wagner et al. 2008). In *D. melanogaster*, for example, highly pleiotropic genes such as ELAV/Hu factors comprise a conserved family of RNA binding proteins, maintain stable expression across neural lineages while also functioning in RNA processing, an interplay that restricts their evolutionary flexibility (Lee et al. 2021). Our observation that pleiotropic genes show similar expression breadth to nonpleiotropic neuronal genes raises additional questions about the underlying regulatory mechanisms that constrain or diversify gene expression. Neuronal genes are often broadly expressed during development due to the complexity of neural tissue formation and function (Crews 2019). This similarity could suggest that neural development shares a demand for versatile gene expression, or alternatively, that some of the pleiotropic genes are particularly important for neurological functions throughout life. Notably, the nonpleiotropic immune gene set shows an enrichment of genes with τ values near 1, indicating a large subset of highly stage-specific immune genes. This pattern is consistent with immune genes that are tightly regulated and expressed predominantly during specific physiological windows, such as acute immune activation or pathogen exposure (Lemaitre and Hoffmann 2007; Buchon et al. 2014). In contrast, pleiotropic genes display fewer extreme τ values, suggesting broader expression across stages, likely reflecting their dual functional roles across systems. The abundance of highly stage-specific immune genes may reduce pleiotropic constraint within the immune-only gene set, allowing more specialized regulation (Alvarez-Ponce et al. 2017), whereas pleiotropic genes remain evolutionarily constrained due to their sustained expression and functional importance across both immune and neuronal contexts.

Genes with stronger evolutionary constraint were more frequently associated with neuronal disease, regardless of whether they were pleiotropic or neuron-specific (Fig. 5). This pattern suggests that strong purifying selection on genes involved in essential neural and immune-related processes, rather than pleiotropy alone, is associated with disease involvement. This was surprising because it contrasts to some extent with previous associations between pleiotropy and disease. For example, Ittisoponpisan et al. (2017) analyzed pleiotropic proteins as those in which variants in the same protein cause multiple distinct diseases and found that these proteins were more likely to be associated with disease, suggesting that genes with multiple functional roles are under stronger functional constraint. It is worth noting that our study links *Drosophila* evolutionary statistics to human ortholog-based disease annotations, and there is no guarantee that selection pressures across these very different taxa have remained consistent over time, despite reasonable conservation of immune and neuronal gene roles. Nevertheless, our results provide an evolutionary perspective that complements the network-based findings of previous work, suggesting that the apparent enrichment of disease associations in pleiotropic genes may be largely explained by their stronger evolutionary constraint rather than pleiotropy itself. Further research with expanded datasets and disorder-specific analyses will help clarify how pleiotropy and evolutionary constraint interact to shape patterns of disease association.

Overall, our findings demonstrate that neuronal pleiotropy slows the evolution of immune genes in a manner similar to previously established constraints imposed by developmental pleiotropy. Neuro-immune pleiotropic genes in *D. melanogaster* evolve more slowly than immune-specific genes, are broadly expressed across life stages, and exhibit evolutionary patterns closely associated with neuronal genes. Importantly, we also show that *Drosophila* genes with human orthologs associated in neurological disorders tend to be those under the strongest purifying selection, underscoring that evolutionary conservation in flies can serve as a useful indicator of disease relevance in humans. Together, these patterns suggest that pleiotropy imposes strong functional constraints on essential neuro-immune genes, limiting their evolutionary flexibility and increasing vulnerability to deleterious mutations. Such constraints may help explain why variants in these genes are repeatedly implicated in neurological disorders, despite strong purifying selection. These two systems are increasingly recognized as highly interconnected through shared signaling pathways and disease mechanisms, making neuro-immune pleiotropy a particularly informative model for studying evolutionary constraint. Future work that distinguishes neurodevelopmental genes from genes functioning primarily in mature neurons may help clarify the relative contribution of these processes to the patterns observed here. Future work integrating population genetic analyses, comparative genomics across additional taxa, and functional assays will further disentangle the selective forces maintaining pleiotropy in immune genes and reveal why some highly constrained neuro-immune genes remain vulnerable points in the architecture of human neurological disease.

## Supplementary Material

jkag223_Supplementary_Data

## Data Availability

All data analyzed in this study are publicly available. Gene IDs and Gene Ontology annotations were obtained from FlyBase (https://flybase.org). RNA-seq expression data were obtained from the modENCODE datasets curated in FlyBase (https://wiki.flybase.org/wiki/FlyBase:ModENCODE_data_at_FlyBase#RNA-Seq_RPKM_Data). Gene Ontology enrichment analyses were performed using PANTHER (http://pantherdb.org). All processed data, including gene lists, biological process counts, dN/dS values, expression specificity (τ) values, and enrichment results, are provided in Supplementary Tables 1 to 12 in Supplementary File 1. Scripts used to analyze the data and generate the figures are available in protocol.io (DOI: https://dx.doi.org/10.17504/protocols.io.rm7vz49d8lx1/v1). Supplemental material available at G3 online.
