# Supplementary material for: Signatures of selection in pleiotropic genes involved in insect neuronal and immune systems
Source: G3 (Bethesda). 2026 Aug 13;16(10):jkag223. doi: 10.1093/g3journal/jkag223 (PMC13643757; doi:10.1093/g3journal/jkag223)
Supplement: Supplementary file 1 [file jkag223_supplementary_data.zip › Figure_S1_G3-2026-406949.pdf]

A

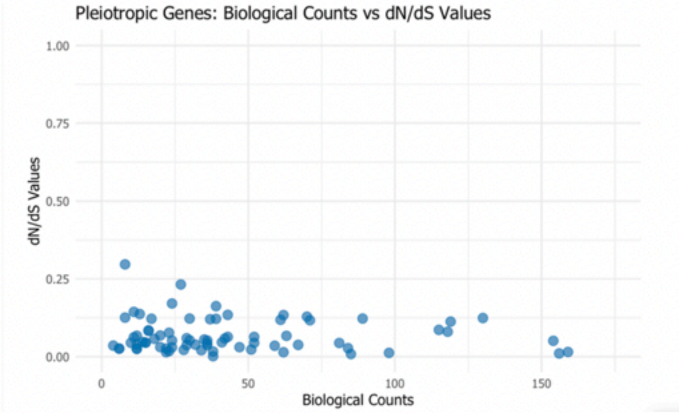
Pleiotropic Genes: Biological Counts vs dN/dS Values
1.00
0.75
0.50
0.25
0.00
dN/dS Values
0
50
100
150
Biological Counts

B

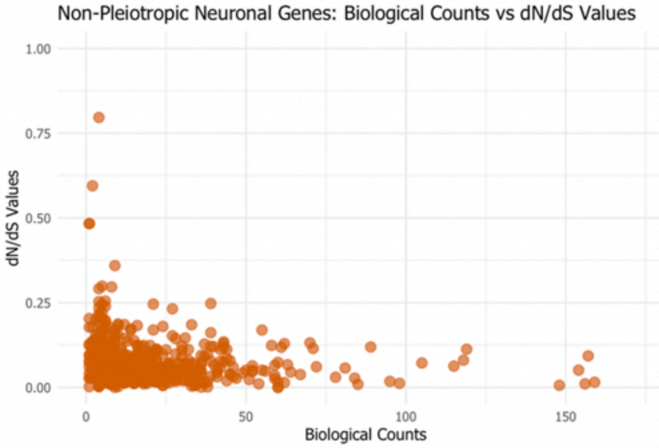
Non-Pleiotropic Neuronal Genes: Biological Counts vs dN/dS Values
1.00
0.75
0.50
0.25
0.00
dN/dS Values
0
50
100
150
Biological Counts

C

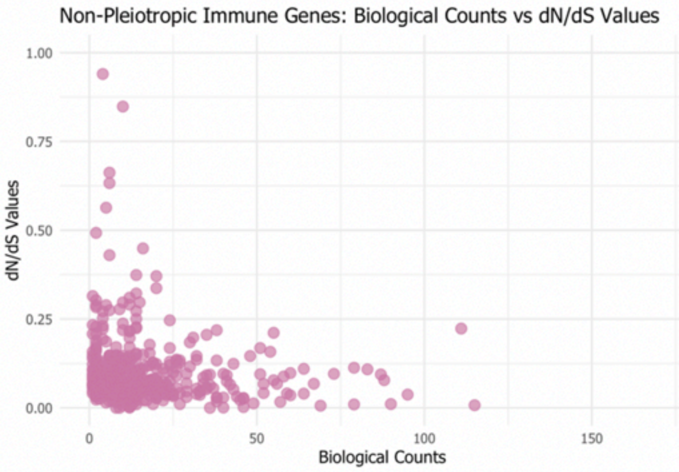
Non-Pleiotropic Immune Genes: Biological Counts vs dN/dS Values
1.00
0.75
0.50
0.25
0.00
dN/dS Values
0
50
100
150
Biological Counts
