# Supplementary material for: Signatures of selection in pleiotropic genes involved in insect neuronal and immune systems
Source: G3 (Bethesda). 2026 Aug 13;16(10):jkag223. doi: 10.1093/g3journal/jkag223 (PMC13643757; doi:10.1093/g3journal/jkag223)
Supplement: Supplementary file 1 [file jkag223_supplementary_data.zip › Figure_S2_G3-2026-406949.pdf]

Top

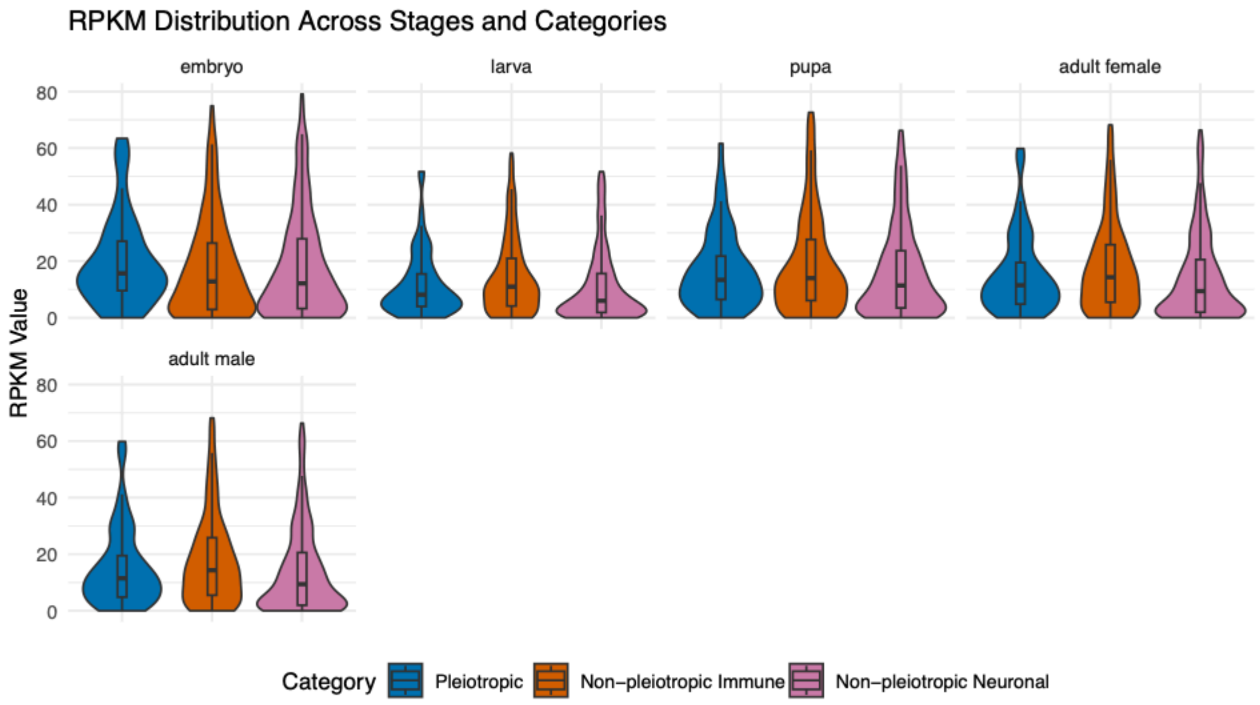

RPKM Distribution Across Stages and Categories
embryo
larva
pupa
adult female
adult male
RPKM Value
0
20
40
60
80
Category
Pleiotropic
Non−pleiotropic Immune
Non−pleiotropic Neuronal

Bottom

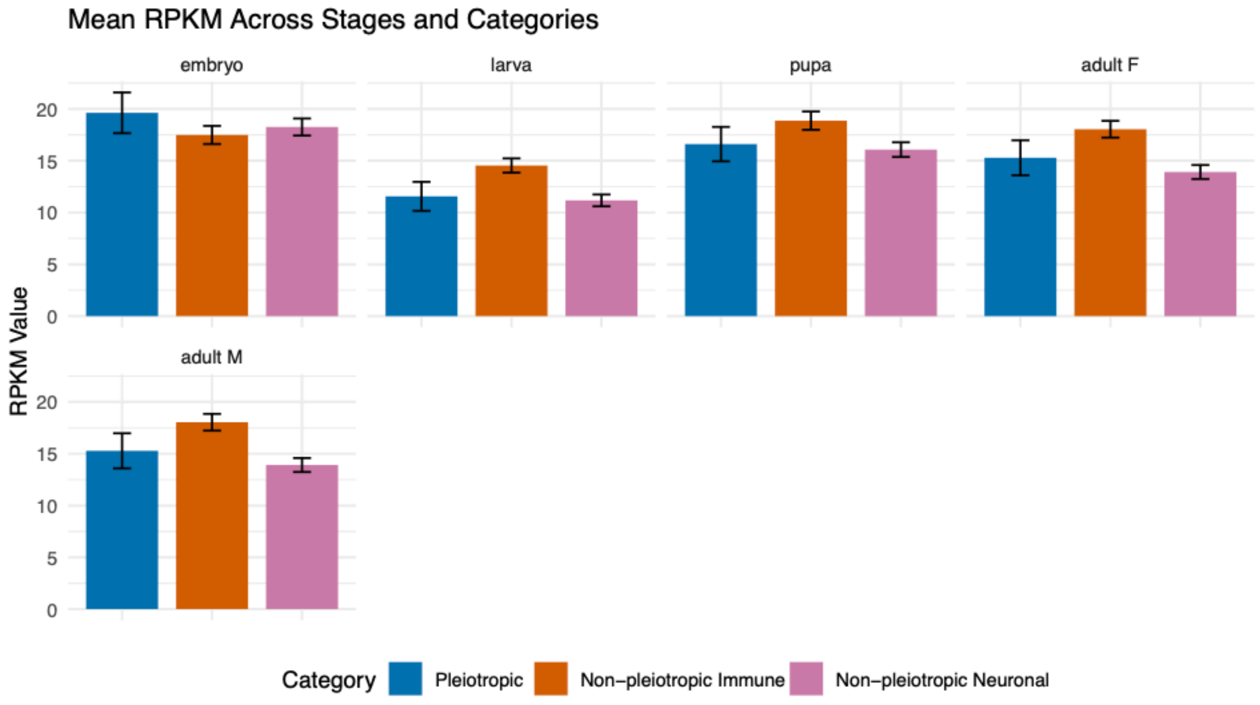

Mean RPKM Across Stages and Categories
embryo
larva
pupa
adult F
adult M
RPKM Value
0
5
10
15
20
Category
Pleiotropic
Non−pleiotropic Immune
Non−pleiotropic Neuronal
