# Supplementary material for: Signatures of selection in pleiotropic genes involved in insect neuronal and immune systems
Source: G3 (Bethesda). 2026 Aug 13;16(10):jkag223. doi: 10.1093/g3journal/jkag223 (PMC13643757; doi:10.1093/g3journal/jkag223)
Supplement: Supplementary file 1 [file jkag223_supplementary_data.zip › Figure_S3_G3-2026-406949.pdf]

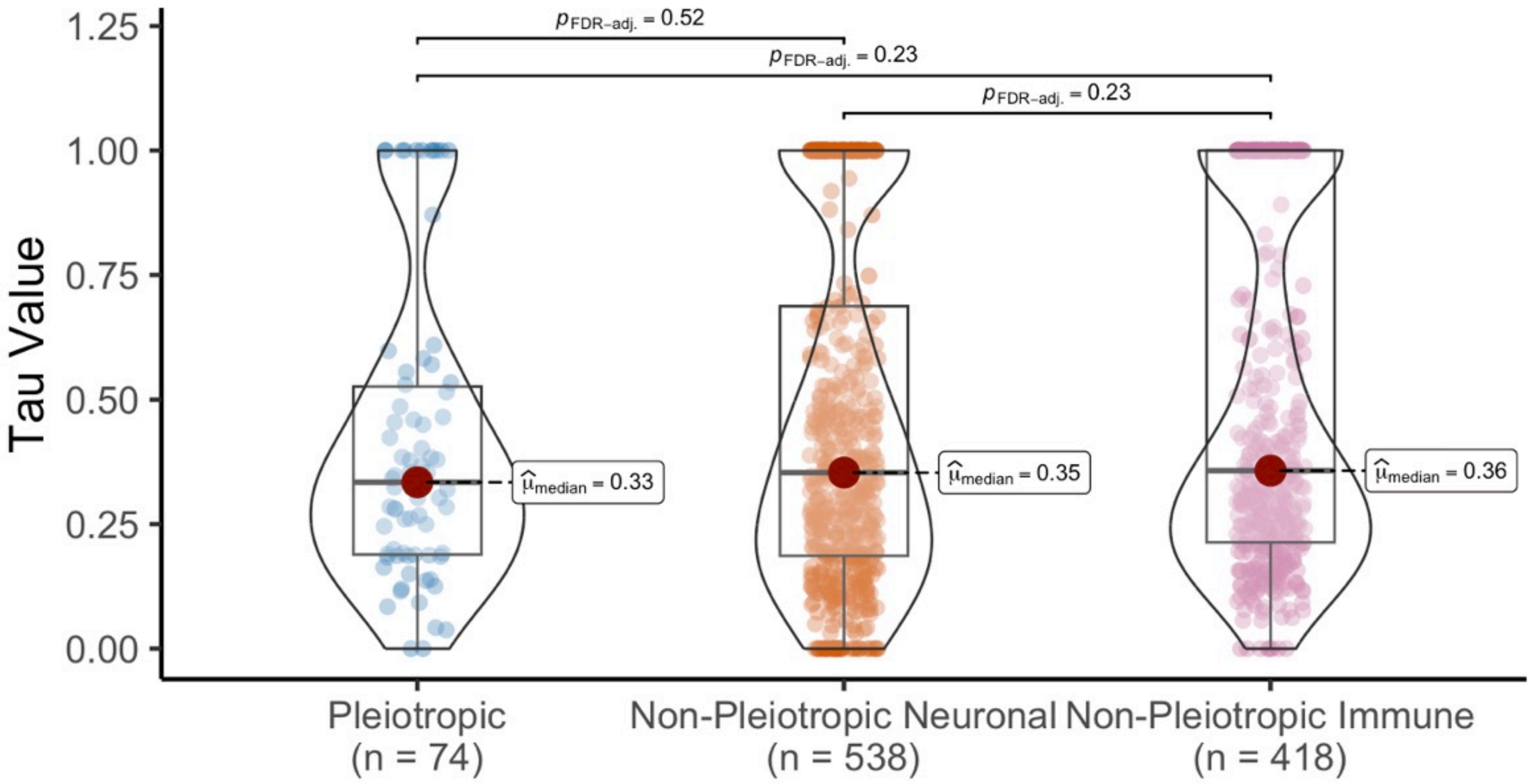

Tau Value
1.25
1.00
0.75
0.50
0.25
0.00
$p_{FDR-adj.}$ = 0.52
$p_{FDR-adj.}$ = 0.23
$p_{FDR-adj.}$ = 0.23
$\hat{\mu}_{median}$ = 0.33
$\hat{\mu}_{median}$ = 0.35
$\hat{\mu}_{median}$ = 0.36
Pleiotropic (n = 74)
Non-Pleiotropic Neuronal (n = 538)
Non-Pleiotropic Immune (n = 418)
