# Supplementary material for: Signatures of selection in pleiotropic genes involved in insect neuronal and immune systems
Source: G3 (Bethesda). 2026 Aug 13;16(10):jkag223. doi: 10.1093/g3journal/jkag223 (PMC13643757; doi:10.1093/g3journal/jkag223)
Supplement: Supplementary file 1 [file jkag223_supplementary_data.zip › Supplemental_Material_Legends_G3-2026-406949.docx]

***Supplemental Material Legends***

***Supplemental Figures***

Figure S1. Relationship between the number of biological counts and individual dN/dS values across gene categories.

Figure S2. Inclusion of embryonic expression increases developmental stage specificity (τ) across gene classes

Figure S3. Stage-specific expression distributions of genes across developmental stages, measured as RPKM values from RNA-Seq data

***Supplemental Tables***

All Tables listed in File S1

Table S1. List of gene IDs for genes binned into pleiotropic, non-pleiotropic immune, and non-pleiotropic neuronal categories

Table S2. Biological process descriptions for genes in each pleiotropy gene class

Table S3. Summary of biological process counts for each gene ID

Table S4. dN/dS ratios for each gene ID

Table S5. RPKM data for genes binned into pleiotropic class

Table S6. RPKM data for genes binned into non-pleiotropic neuronal class

Table S7. RPKM data for genes binned into non-pleiotropic immune class

Table S8. Average RPKM values for each gene ID

Table S9. Tau values for each gene ID

Table S10. GO enrichment analysis for pleiotropic genes

Table S11. GO enrichment analysis for non-pleiotropic neuronal genes

Table S12. GO enrichment analysis for non-pleiotropic immune genes

Table S13. Logistic regression model predicting neurological disease association statistics
